# Supplementary material for: The causal relationship between the human gut microbiota and pyogenic arthritis: a Mendelian randomization study
Source: Front Cell Infect Microbiol. 2024 Nov 26;14:1452480. doi: 10.3389/fcimb.2024.1452480 (PMC11629706; doi:10.3389/fcimb.2024.1452480)
Supplement: Supplementary file 1 [file DataSheet1.zip › mendelian test/ebi-a-GCST90027760.csv_leave_one_out.pdf]

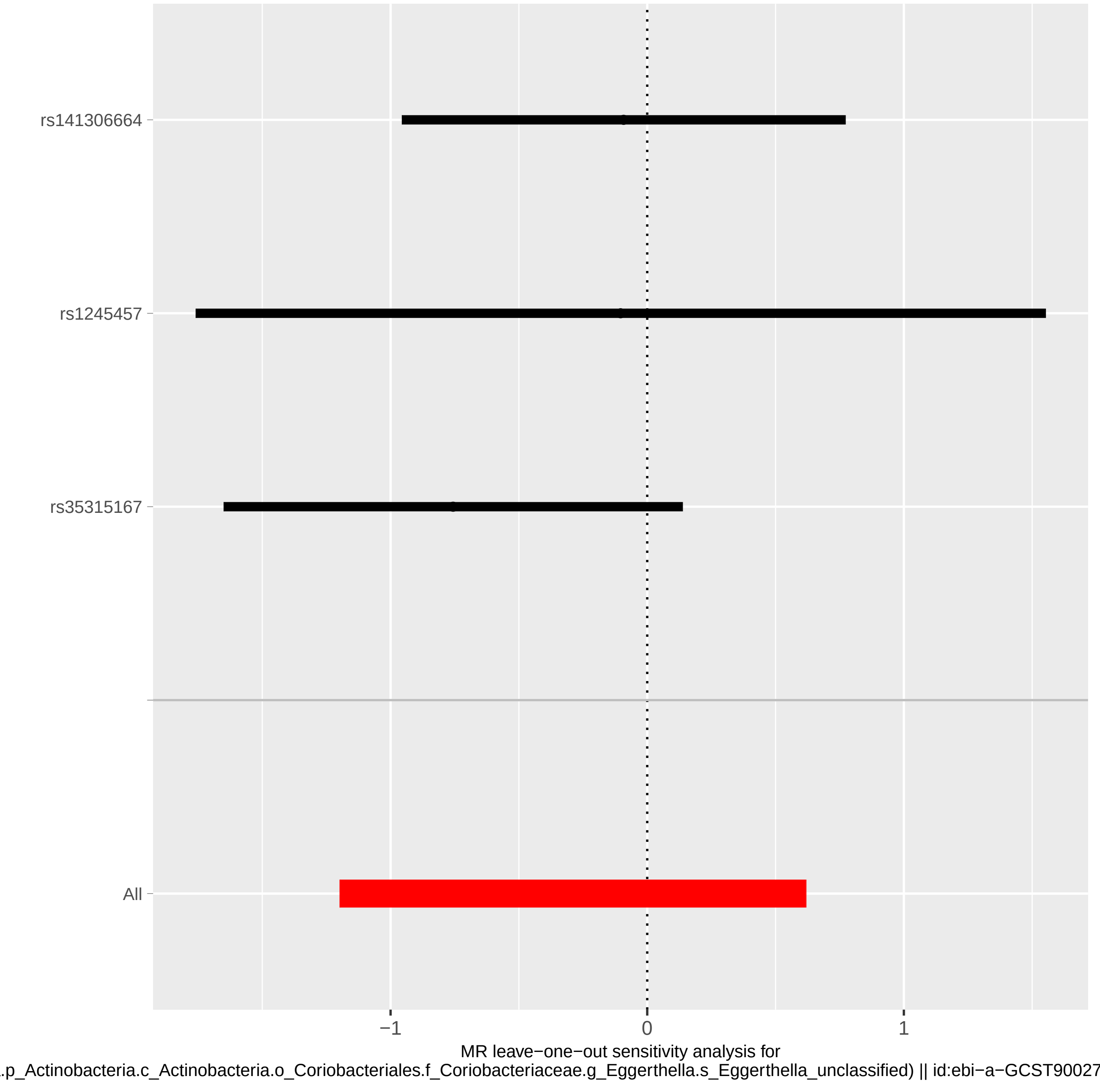
rs141306664
rs1245457
rs35315167
All
−1
0
1
MR leave−one−out sensitivity analysis for
.p_Actinobacteria.c_Actinobacteria.o_Coriobacteriales.f_Coriobacteriaceae.g_Eggerthella.s_Eggerthella_unclassified) || id:ebi−a−GCST90027
